# A rare case of pelvic primitive neuroectodermal tumor with misleading symptoms: A case report

**DOI:** 10.1016/j.amsu.2020.05.010

**Published:** 2020-05-16

**Authors:** Kusay Ayoub, Ammar Niazi, Baraa Shebli, Rand Batal, Hamed Kozom, Lina Ghabreau, Nihad Mahli

**Affiliations:** a Department of Surgery, Aleppo University Hospital, University of Aleppo, Syria; b Faculty of Medicine, University of Aleppo, Syria; c Department of Pathology, Aleppo University Hospital, University of Aleppo, Syria

**Keywords:** Primitive neuroectodermal tumors, Surgical oncology, Case report, pPNET, Peripheral Primitive NeuroEctodermal Tumors, ES, Ewing Sarcoma, PNET, Primitive NeuroEctodermal Tumors

## Abstract

Peripheral Primitive NeuroEctodermal Tumors (pPNETs) are rare highly malignant tumors; originating from the neuroectoderm. Although PNETs may arise in various locations (most commonly in the extremities), very few cases have been reported in the pelvis. There is still poor evidence about the management of these tumors in the literature.

We present a rare case of pelvic PNET in a 20-year-old male. The patient presented with symptoms mimicking a lumbar disk hernia, which delayed the diagnosis. He was managed with a combination of a debulking procedure, adjuvant chemotherapy, radiotherapy; and has been in remission for 2 years upon follow-up.

This case highlights the importance of diagnosing such aggressive tumors as early as possible (as prognosis may vary significantly), and the challenge in the management of PNETs due to poor evidence.

## Highlights

• Primitive NeuroEctodermal Tumors (PNET) are highly malignant tumors that generally manifest in the extremities.
• Clinical symptoms are usually due to mass effect and depend on the site of presentation, but can sometimes be misleading.
• There is still poor evidence about the management of PNET in the existing literature.

## 1 Introduction

This case has been reported in line with the SCARE criteria [1]. Ewing Sarcoma/Primitive NeuroEctodermal Tumors (ES/PNETs) are malignant tumors that originates from the neuroectoderm [2].

In 1996, Batsakis et al. divided PNETs into 3 groups based on the tissue of origin: peripheral PNET, central nervous system PNET and neuroblastoma [3]. Peripheral primitive neuroectodermal tumors (pPNETs) manifest in various locations, including kidney, bone, face, and soft tissue [4]; but they have a marked predilection for the extremities [5]. The pelvic localization of PNETs is very rare (Yan et al. reported 2 cases out of 36 PNETs) [6]. In this report, we describe a rare case of PNET in the left iliac fossa highlighting the clinical manifestations, management and follow up.

## 2 Case presentation

A 20-year-old male presented to the clinical center in the village with a complaint of pain in the lower back spreading over the left lower limb with paresthesia mimicking a lumbar disk hernia. He was given analgesic drugs that eased the pain several weeks.

**Fig. 1:**
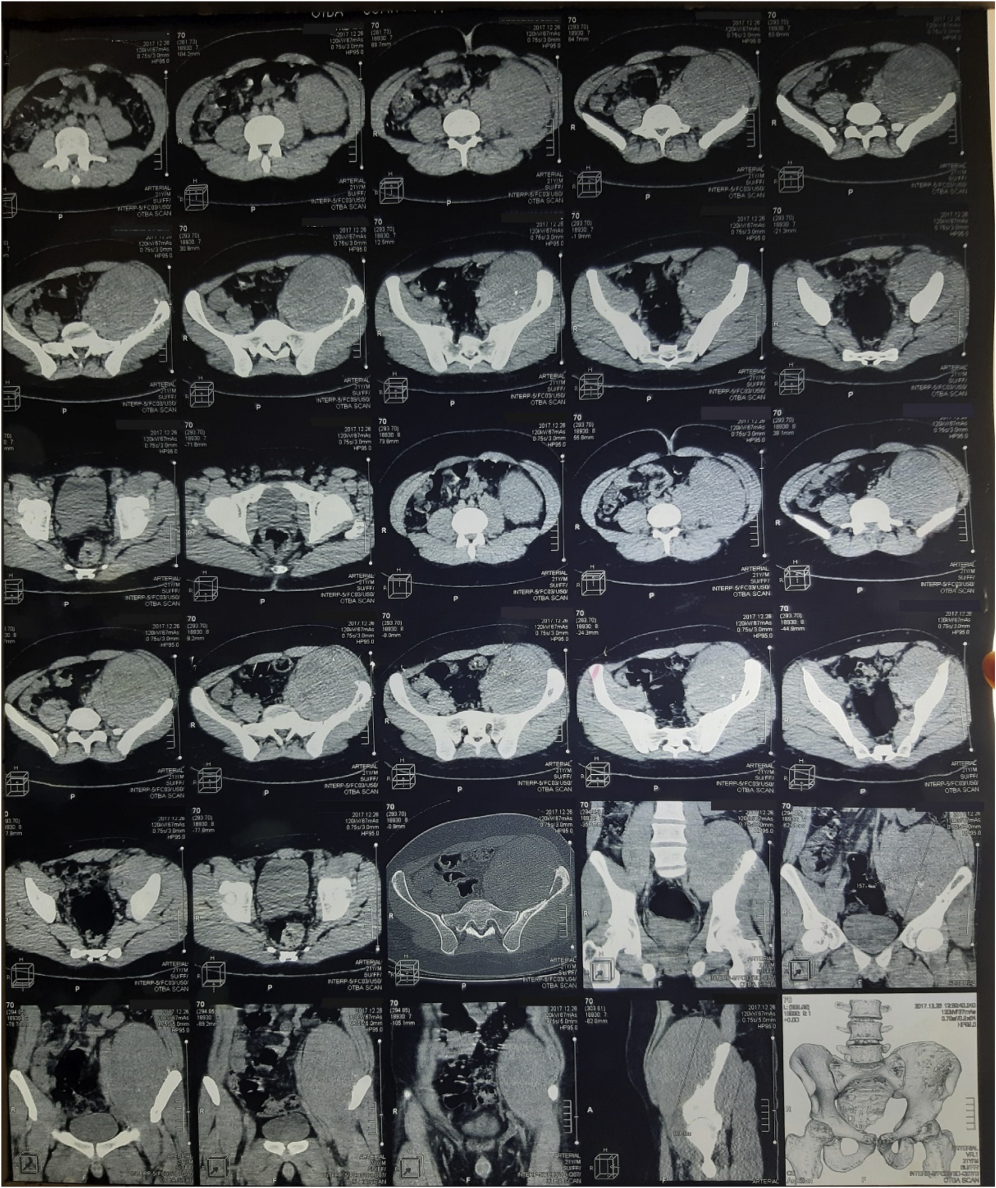
A CT scan showing a huge mass in the left iliac fossa.

The patient then noticed a mass bulging from the left lower quadrant of the abdomen and was referred to the hospital by the family physician. He didn't have any other symptom or a significant past medical history. Familial history included hypertension and diabetes mellitus. Physical examination showed a mass in the left iliac region. A CT scan showed a huge lytic mass (longest diameter: 19 cm) occupying the left iliac fossa and pushing the adjacent structures. Some erosion was evident in the iliac crest (see Fig. 1).

The laboratory tests showed an elevated ESR (68 mm/h), elevated INR (1.85), low albumin levels (2.5 mg/dl) with otherwise normal values.

**Fig. 2:**
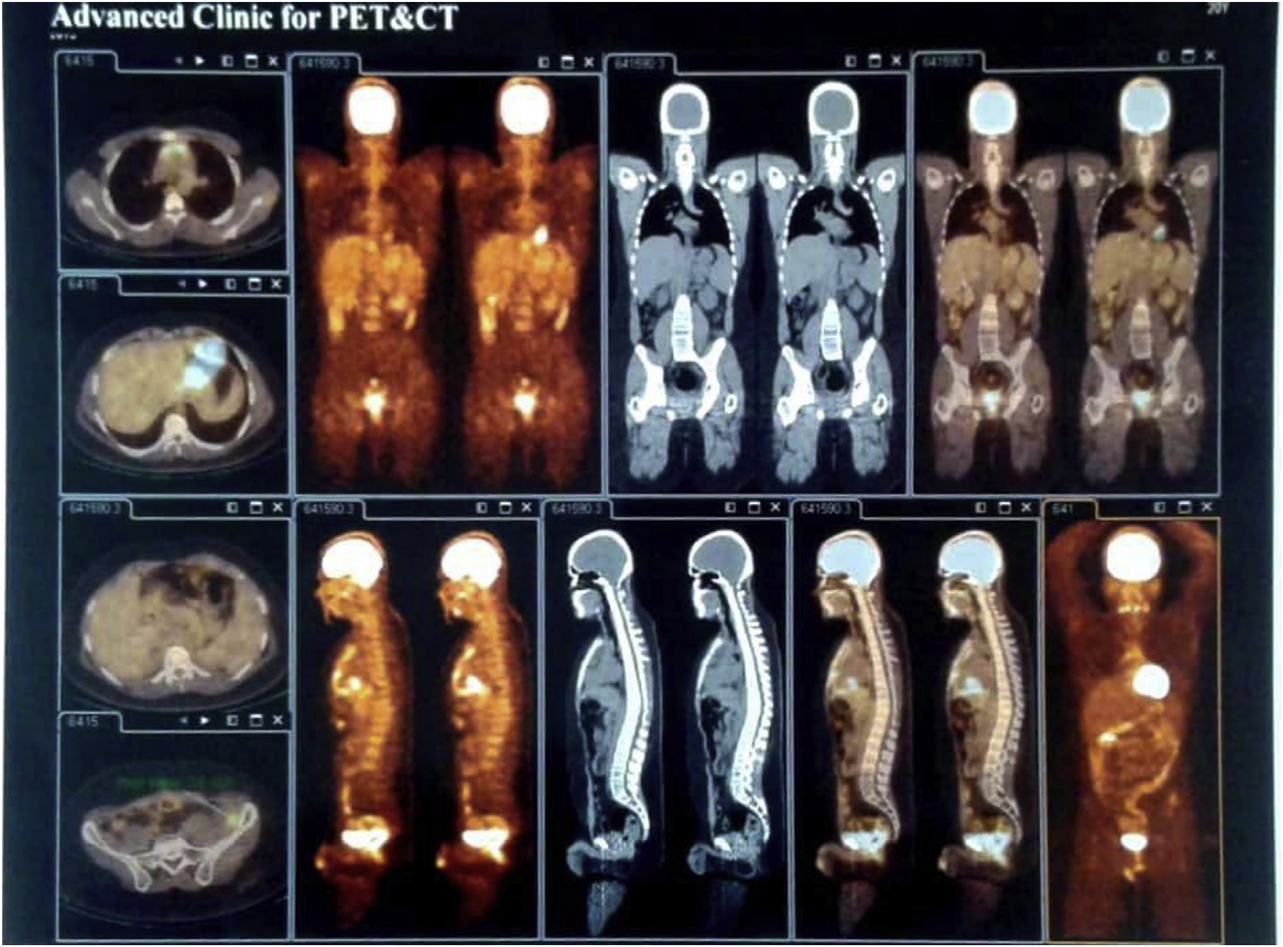
A multislice CT & PET scan after treatment showing small active remnants around the iliac without any other any involvement.

The patient refused to perform a biopsy, so an instructor surgeon in the general surgery department performed an exploratory laparotomy. The tumor infiltrated the local structures and the spinal nerves (which explains the previous presentation), so a complete resection was not possible. We performed a debulking procedure of the tumor and sent a biopsy to the pathology. The tumor was so infiltrative that we couldn't specify whether it originated from the iliac crest or from the retroperitoneal soft tissue (see Fig. 2).

The microscopic examination showed malignant proliferation of solid nests and cords of small compact oval and round tumor cells, with wide areas of necrosis. A final diagnosis needed an immunstaining. Current Stains were used: CD99, LCA, CD79 and CD34. CD99 was positive and the final diagnosis was Primitive NeuroEctodermal Tumor (PNET).

We consulted the oncology department and transferred the patient to adjuvant therapy; which included a combination of chemotherapy and radiotherapy. The patient took 4 sessions of chemotherapy (mesna, etoposide and cyclophosphamide) followed by 50 Gy of radiotherapy divided to 25 sessions, each of 2 Gy.

After the completion of the treatment, a multislice PET/CT scan showed a few small active remnants around the iliac bone without any nodal or visceral involvement.

**Fig. 3:**
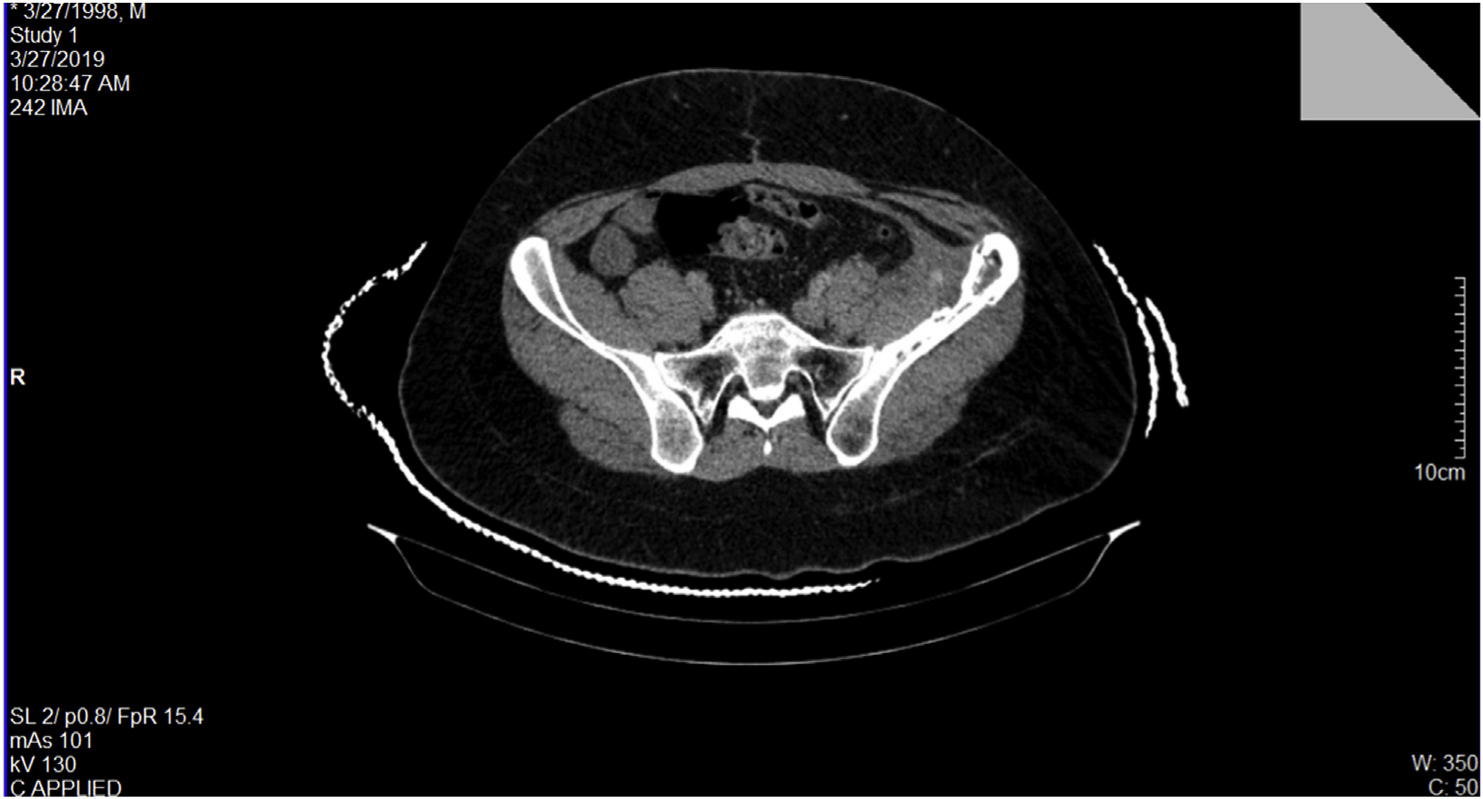
The last follow-up CT scan showing the residual erosion of the iliac crest without any evolvement of the tumor.

The patient is followed up by CT scan every 4 month. The last follow up CT scan, after two years of diagnosis, showed complete remission without any evolvement of the tumor compared to the previous images (see Fig. 3).

## 3 Discussion

PNETs are a group of rare tumors with an annual incidence of 2.1 cases per million children [2]. PNETs are divided into 3 groups: peripheral PNET, central nervous system PNET and neuroblastoma [3]. pPNETs are most common in deep soft tissues of thigh and buttocks (extremities) followed by the paravertebral region, abdomen, head and neck region, thoracopulmonary region (Askin tumor), etc. . Only a few cases of pelvic PNETs have been reported until now [5].

Patients generally present with rapidly enlarging masses and symptoms related to tumor mass effects [7]. In our case, a 20-year-old male presented with misleading symptoms of lower back pain spreading over the left lower limb and paresthesia; a bulging mass supervened two months later.

PNETs are very aggressive tumors, sometimes presenting with metastatic disease [5]. Luckily, we were able to diagnose it before the tumor became metastatic, but it was very infiltrative that we could not specify its origin. Metastatic spread worsens patient's prognosis and increase mortality rates [8].

We did a microscopic examination that showed malignant proliferation of tumor cells. Immunohistochemical examination showed a positive CD99 similar to the microscopic findings in many other studies confirming the diagnosis [6,9].

PNETs' large size, metastases, poor radiosensitivity and incomplete surgical resection (due to the infiltration) altogether make the prognosis unfavorable [10,11]. This requires multimodal therapy including surgery, radiotherapy, or both combined with multiagent chemotherapy regimens. The combination of vincristine, actinomycin D, cyclophosphamide, and doxorubicin and that of ifosfamide and etoposide appear to offer the best survival chance [4], but even after that, the prognosis is still poor. In our case, a combination of cyclophosphamide, etoposide and mesna was used along with incomplete resection and radiotherapy; ultimate control was achieved and the patient has been in remission. We reported a management protocol alongside one of the longest uneventful follow-up periods. However, closer and longer follow-up is necessary, to see if there will be any relapse or development of metastatic spread.

## 4 Conclusion

Despite their rarity, PNETs should be suspected when CT images show a large, irregular, aggressive cystic-solid mass. Due to the poor evidence in the literature, the management of these tumor needs further research. The multimodal therapy used in the management of our patient achieved full control and remission of the tumor upon 2 years of follow-up and we encourage physicians to consider this protocol when dealing with similar cases.

## Patient consent

Informed consent was obtained from the patient for publication of this case report and accompanying images. A copy of the written consent is available for review by the Editor-in-Chief of this journal on request.

## Provenance and peer review

Not commissioned, externally peer reviewed.

## Funding

There are no funding sources.

## Declaration of competing interest

The authors declare that there is no conflict of interest.
